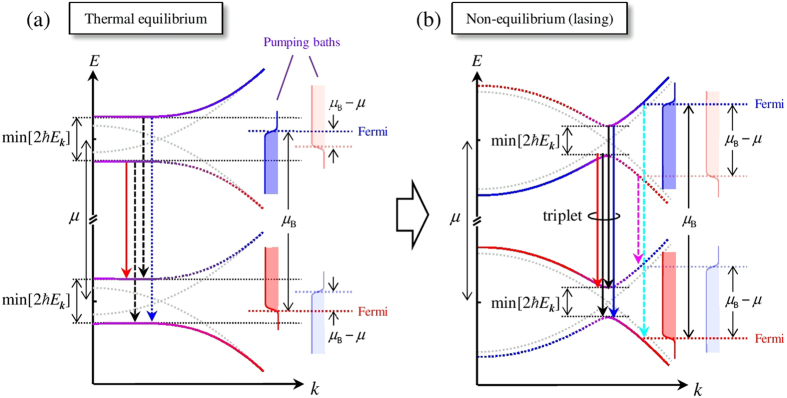# Erratum: High-energy side-peak emission of exciton-polariton condensates in high density regime

**DOI:** 10.1038/srep35094

**Published:** 2016-11-02

**Authors:** Tomoyuki Horikiri, Makoto Yamaguchi, Kenji Kamide, Yasuhiro Matsuo, Tim Byrnes, Natsuko Ishida, Andreas Löffler, Sven Höfling, Yutaka Shikano, Tetsuo Ogawa, Alfred Forchel, Yoshihisa Yamamoto

Scientific Reports 6: Article number: 2565510.1038/srep25655; published online: 05 19 2016; updated: 11 02 2016

This Article contains an error in Figure 3(B), where the Fermi distributions of the pumping path are incorrectly shaded black. The correct Figure 3 appears below as Figure 1.

## Figures and Tables

**Figure 1:**