# Supplementary material for: High-energy side-peak emission of exciton-polariton condensates in high density regime
Source: Sci Rep. 2016 May 19;6:25655. doi: 10.1038/srep25655 (PMC4872130; doi:10.1038/srep25655)
Supplement: Supplementary Information [file srep25655-s1.doc]

**Supplementary Information**

High-energy side-peak emission of exciton-polariton condensates in high density regime

Tomoyuki Horikiri^1,2,3,4,5^, Makoto Yamaguchi^4,6^, Kenji Kamide^3,6^, Yasuhiro Matsuo^1,3^, Tim Byrnes^7,8,1^, Natsuko Ishida^4^, Andreas Löffler^9^, Sven Höfling^9,10,1^, Yutaka Shikano^11,12,13^, Tetsuo Ogawa^6,14^, Alfred Forchel^9^, and Yoshihisa Yamamoto^1,2,3,4,15^

Correspondence to: [yyamamoto@stanford.edu](mailto:yyamamoto@stanford.edu), horikiri@ynu.ac.jp

**This file includes:**

Mollow triplet — similarities and differences

Comparisons with a two-level model

Theoretical treatments

Figs. S1 to S9

**S1: Mollow triplet — similarities and differences**

In quantum optics, it is well known that a spectral triplet can be observed in the fluorescence spectrum from a two-level atom driven by a resonant continuous-wave laser field. Such a triplet is called the Mollow triplet^23^, and it has close relations to the spectra discussed in our study. Therefore, in this section, the similarities and differences between them are presented.

First, we briefly review the mechanism and the spectral properties of the Mollow triplet in atomic systems. When a laser field is applied to a single two-level atom, dressed states can be formed as a result of the strong coupling between the atom and photon states. Such dressed states are described by

$|\pm,n\rangle=\frac{1}{\sqrt{2}}\left[|\text{excited},n-1\rangle\pm|\text{ground},n\rangle\right]$, (S1)

where *n* is the photon number and |ground⟩ (|excited⟩) is the ground (excited) state of the atom. The eigenenergies of these states are written as

, (S2)

where *ω*_0_ is the laser frequency and $\Omega_{n}=2g\sqrt{n+1}$ is the Rabi frequency. Therefore, in the strong-field limit (*n* ≫ 1), Ω*_n_* ≈ Ω*_n_*_+1_ ≡ Ω_R_ is a good approximation, and four transitions become possible between the eigenstates (see Fig. S1A). As a result, three peaks are formed in the fluorescence spectrum. We note that the energy separation between the main peak and the sideband peaks, which corresponds to Ω_R_, is roughly proportional to the square root of the intensity of the incident laser (that is, it is roughly proportional to ) in the strong-field limit. Such a fluorescence spectrum *S*(*ω*) can be written as

$$S\left(\omega\right)=\frac{I}{2\text{π}}\frac{\text{π}}{\gamma^{2}+2\Omega_{\text{R}}^{2}}\delta\left(\omega-\omega_{0}\right)$$

$+\frac{I}{2\text{π}}\left\{\frac{1}{2}\frac{\gamma/2}{[\gamma/2]^{2}+[\omega-\omega_{0}]^{2}}+\frac{1}{4}\frac{3\gamma/4}{[3\gamma/4]^{2}+[\omega-[\omega_{0}+\Omega_{\text{R}}]]^{2}}+\frac{1}{4}\frac{3\gamma/4}{[3\gamma/4]^{2}+[\omega-[\omega_{0}-\Omega_{\text{R}}]]^{2}}\right\}$, (S3)

where *I* is a constant value and *γ* is the relaxation rate of the atom. The first term is the elastic Rayleigh scattering of the laser field, and the last three terms correspond to the fluorescence spectrum. Here, the Rabi frequency Ω_R_ can be made larger than the order of *γ* by using a sufficiently strong laser field. Hence, the spectra from the last three terms are well separated, resulting in the Mollow triplet, while the first elastic scattering term becomes negligible for Ω_R_ ≫ *γ*. From this expression, we can obtain the following spectral properties: the central peak at *ω*_0_ has a linewidth of
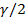
, and the peak height is three times larger than the side peaks, while the side peaks of the frequencies *ω*_0_ ± Ω_R_ have linewidths of 3*γ*/4. In contrast, in the weak field limit (Ω_R_ ≪ *γ*), *S*(*ω*) shows a single-peak spectrum located at *ω*_0_. In what follows, this is referred to as a simple Mollow triplet to emphasize the conventional physical picture.

The simple Mollow triplet also appears in emission spectra when the atom is strongly coupled to a single-mode cavity^24^. In this case, the triplet can be found when a strong coherent light field is initially present inside the cavity. However, the vacuum Rabi splitting can be found when the coherent light field becomes sufficiently weak because the atom is still strongly coupled to the vacuum photon state of the cavity (see Fig. S1B). As a result, a smooth change from the vacuum Rabi splitting to the simple Mollow triplet can be seen in the emission spectra when the atom is strongly coupled to a cavity^24^.

In the exciton–polariton case, Mollow-triplet-like spectra are also theoretically predicted, first in the equilibrium limits^1^ and then in the non-equilibrium cases^19^ where the side peaks are called amplitude modes and the excitons are simply taken as two-level systems. In the present work, the PL, not only in the equilibrium case but also in the non-equilibrium case, has been calculated by explicitly treating the semiconductor electrons and holes with their Coulomb interactions (see also Section 3 for the calculation methods). The spectral features in this case are not completely the same as those of the simple Mollow triplet because of, e.g., the dispersions of electrons and holes and the many-body effects^31,32^, but the physical picture analogous to the Mollow triplet is still useful for understanding their emission mechanisms. The typical energy diagrams in a high-excitation regime are shown in Fig. S2. The energy dispersions of the electrons and holes undergo the band-gap renormalization (BGR) when only the electron–electron and hole–hole Coulomb interactions are considered (Fig. S2A). Then, the energy gap of min[2*ħE_k_*] ~ 2*ħ*Δ appears in each band, as shown in Fig. S2B, if we also switch on the dipole coupling to the cavity photons and the electron–hole (e–h) attractive Coulomb interaction. The formal definitions of the minimum e–h pair breaking energy min[2*ħE_k_*] and the generalized Rabi frequency Δ*_k_* can be found in Subsection S3.2. Here, the energy gap is formed not only by the standard Rabi splitting due to the strong light field inside the cavity^24^ but also by the BCS-like e–h Coulomb correlations^22,26-28^. Thus, the spectral triplet can also be predicted in the semiconductor model.

However, unlike the simple Mollow triplet, the intensity ratio of the side peaks can change, depending on the steady-state conditions. In the case of thermal equilibrium, the energy needed to break e–h pairs, min[2*ħE_k_*], is larger than *μ*_B_ – *μ* + 2*ħγ* + 2*k*_B_*T* (see Ref. 22 and also Subsection S3.2), a typical example of which is shown in Fig. S3A. Here, *μ*_B_, *μ,* 1/*γ*, and *T* represent the chemical potential of the electron and hole pumping baths (which we refer to as the pumping parameter in the main text, since it measures the degree of pumping), the oscillation frequency of the photon and polarization fields, the thermalization time scale of the pumping baths, and the temperature defined in the pumping baths. In this case, the Fermi surface of the pumping baths cannot go beyond the energy gap. As a result, the lower-energy peak (indicated by the solid red arrow) becomes large because sufficient electrons and holes can be obtained from the pumping baths to recombine and emit photons into the low-energy peak. However, the situation is opposite for the high-energy peak (the dotted blue line); electrons and holes are not supplied by the pumping baths. Hence, the low-energy peak becomes larger than the high-energy peak in the thermal equilibrium regimes. In contrast, non-equilibrium effects cannot be negligible when *μ*_B_ – *μ* becomes larger than min[2*ħE_k_*] – 2*ħγ* – 2*k*_B_*T*, and there are ***k***-regions described by the Maxwell–semiconductor–Bloch equations (MSBE) used in semiconductor lasers when *μ*_B_ – *μ* ≳ min[2*ħE_k_*] + 2*ħγ* + 2*k*_B_*T* (see also Subsection S3.2). In this case, the Fermi surface of the pumping baths exceeds the energy gap min[2*ħE_k_*], a typical example of which is shown in Fig. S3B. Hence, the high-energy peak can become stronger than in the thermal equilibrium cases, which is significant since thermal equilibrium can cause fairly strong high-energy peaks.

Furthermore, to understand the emission mechanism of exciton–polariton microcavity systems, the important role that many-body effects play must also be considered. In semiconductor materials (with no cavity), it is well known that e–h Coulomb interactions cause significant enhancement of the PL intensity around the Fermi energy even for a plasma state^31,32^. This effect also should be treated in our case, and as a result, the high-energy peak is further enhanced when the Fermi energy (which is given by *μ*_B_) roughly coincides with the high-energy peak located at *μ* + min[2*ħE_k_*]. In fact, in our calculations, the intensity of the high-energy peak exceeds that of the low-energy peak only when *μ*_B_ – *μ* ~ min[2*ħE_k_*]. In this sense, it is clear that the many-body effects also play an essential role, which is absent in, e.g., the two-level model (see also Section S2). It is instructive to note that the condition *μ*_B_ – *μ* ~ min[2*ħE_k_*] is equivalent to the crossover condition from quasi-equilibrium phases (*μ*_B_ – *μ* ≲ min[2*ħE_k_*]) into lasing phases (*μ*_B_ – *μ* ≳ min[2*ħE_k_*]) when ignoring the effects of *γ* and *T* ^16^. Therefore, we can conclude that the system is near the crossover regime into lasing if the high-energy peak is stronger than the low-energy peak, at least in principle.

**S2: Comparisons with a two-level model**

In the present work, the theoretical discussions are based on the framework explained in the next section (Section S3), where electrons and holes are explicitly treated with their Coulomb interactions and the many-body effects resulting from the Coulomb interactions are included within the Hartree–Fock (HF) level. However, it is important to compare the results with another model where the e–h system is simply treated as a set of two-level systems (TLSs)^19,43,44^. Hence, in this section, we present numerical results based on such a TLS model and show that the height of the high-energy peak does not exceed that of the low-energy peak. We note that some results include new viewpoints on the result that the quasi-equilibrium and non-equilibrium regimes are clearly distinguished even for the TLS model.

The model discussed here is schematically shown in Fig. S5 with the *n* two-level systems. In our calculations, the cavity is resonant with the transition energy *ħω*_x_, and the lower polariton energy *E*_LP_ is located = 10 meV below the bare cavity, where the coupling constant between the two-level atom and the cavity mode are assumed to be *ħg* = 1.0 meV and *n*= 100, respectively. In addition, *T* = 10 K, *ħκ* = 100 μeV, and *ħγ* = 4 meV are used, which are the same as the parameters used in the main text (see also Subsection S3.5) with the decay rate from the cavity mode *κ*.

Figure S6A shows the calculated photon number inside the cavity (= |*a*_0_|^2^), in the same manner as Fig. 2B in the main text. The number of photons calculated with the thermal equilibrium theory (black solid line: *κ* = 0, *γ* = 0^+^) diverges when *μ*_B_ approaches the bare cavity energy *E*_cav_ for the same reasons as in Fig. 2B. In contrast, the number of photons does not diverge for the non-zero *κ* and *γ*. Here, red data points and lines are used in the plots when the system can still be regarded as in a quasi-equilibrium state, and otherwise blue plot elements are used. The typical emission spectra for quasi-equilibrium and non-equilibrium regimes are shown in Fig. S6, B and C. In the case of quasi-equilibrium, the low-energy peak is stronger than the high-energy peak, which is qualitatively the same as is shown in Fig. 2C. Then, the high-energy peak becomes stronger when the system enters the non-equilibrium regime (Fig. S6C). However, the high-energy peak does not exceed the low-energy peak, which is in stark contrast to the semiconductor model where the Coulomb interactions are explicitly taken into account. As described in Section S1, BCS-like Coulomb correlations also play an important role in the intensity of the high-energy peak in the semiconductor model. However, such a mechanism does not work in the TLS model because there is no Coulomb interaction. As a result, the high-energy peak becomes stronger than in the quasi-equilibrium cases but cannot exceed the low-energy peak in the TLS model. This clearly demonstrates that the many-body effects resulting from Coulomb interactions are indispensable for understanding why the high-energy peak is stronger than the low-energy one. Thus, the results from the TLS model also support the idea that the many-body effects have a strong influence on the intensity of the high-energy peak.

**S3: Theoretical treatments**

In our study, we have employed the recently developed formalism described in Ref. 11 in order to investigate the equilibrium and non-equilibrium natures of the polariton condensate in high-density regimes (for a brief introduction, see also Ref. 17). In this treatment, a closed set of equations for the polarization function *p****_k_***, the number of electrons *n*_e_,***_k_***, the number of holes *n*_h_,***_k_***, and the cavity photon field *a*_0_ is analyzed for the steady state. The advantage of this approach is the applicability to high-density regimes because electrons and holes are explicitly treated with their Coulomb interactions. This is in contrast to the other approaches based on the non-equilibrium Gross–Pitaevskii (GP) equation. Although the GP equation has many good points and enables intuitive and simple analyses^45,46^, the exciton–polaritons are just taken as interacting bosons, which is a model that is only applicable to the low-density regimes. As a result, the fermionic features of the electrons and holes are completely neglected. On the other hand, our approach can be used to study the BEC–BCS–LASER crossover without any limit on the density. Furthermore, the conditions for the system to be well described by quasi-equilibrium theories can also be obtained explicitly. Hence, the formalism is quite helpful for the present work. However, how to discuss the emission spectra from the cavity is not presented in Ref. 22. Therefore, first, the formalism described in Ref. 22 is briefly reviewed in Subsections S3.1 and S3.2, and then, Subsections S3.3 and S3.4 explain how to calculate the emission spectra, which is an extension of the work for two-level systems^19,433,444^. Finally, the numerical procedures and parameters for our calculations are summarized in Subsection S3.5.

S3.1: Model and Hamiltonians

Let us recapitulate the relevant model and Hamiltonians. The model of the electron–hole–photon (e–h–p) system is shown in Fig. S7, where the reservoir responsible for the photonic leakage is the free-space vacuum fields (photon bath) and those for pumping are the pumping baths, which inject (extract) electrons and holes into (from) the e–h system on a time scale of 1/*γ* by emulating the thermalization processes of the e–h system.

The total Hamiltonian in the model is , where

, (S4)

, (S5)

, (S6)

are the system and reservoir Hamiltonians and their interaction Hamiltonian, respectively. Here, and denote annihilation operators for electrons with wavenumber ***k*** in the conduction (c) and valence (v) bands, respectively, and is an annihilation operator for photons with wavenumber ***q*** in the cavity. Similarly, and denote fermion annihilation operators of pumping baths, and $\hat{\Psi}_{\mathbf{p}}$ is the boson annihilation operator of free-space vacuum fields. Here, and in Eq. (S5) are defined as

, , (S7)

where *ħ*^-1^*μ* is the oscillation frequency of the coherent photon field^22^, which will be determined later. $\Gamma_{\mathbf{k}}^{\alpha}$ and $\zeta_{\mathbf{k}}$ in Eq. (S6) are the coupling constants between the system and each reservoir, satisfying the relations

$\text{π}\left|\Gamma_{\mathbf{k}}^{\alpha}\right|^{2}D_{\alpha}^{\text{B}}\left(\omega\right)\cong\gamma_{\alpha,\mathbf{k}}\cong\gamma$, $\text{π}\left|\zeta_{\mathbf{k}}\right|^{2}D_{\text{ph}}^{\text{B}}\left(\omega\right)\cong\kappa_{\mathbf{k}}\cong\kappa$, (S8)

with the following definitions of the density of states:

$D_{\alpha}^{\text{B}}\left(\omega\right)\equiv\sum_{\mathbf{k}} \delta\left(\xi_{\alpha,\mathbf{k}}^{\text{B}}-\omega\right)$, $D_{\text{ph}}^{\text{B}}\left(\omega\right)\equiv\sum_{\mathbf{p}} \delta\left(\xi_{\mathbf{p}}^{\text{B}}-\omega\right)$. (S9)

Here, the dependence on the wavenumber is neglected in Eq. (S8) for simplicity. $\hat{H}_{\text{kin}}$, $\hat{H}_{\text{el-el}}$, and $\hat{H}_{\text{el-ph}}$ in Eq. (S4) are described as

, (S10)

, (S11)

, (S12)

where

, , (S13)

which are similar to the definitions in Eq. (S7). Here, *ω*_c_*,****_k_***, *ω*_v_*,****_k_***, and *ω*_ph_*,****_q_*** denote the conduction-band, valence-band, and photonic dispersion relationships, respectively. In addition, *U'****_q_*** denotes the Coulomb interaction defined as

$U'_{\mathbf{q}}\equiv\left\{\begin{matrix} U_{\mathbf{q}} \\ 0 \end{matrix} \begin{matrix} \text{for} \\ \text{for} \end{matrix}\right. \begin{matrix} \mathbf{q}\neq0 \\ \mathbf{q}=0 \end{matrix}$. (S14)

We note that the Hamiltonian presented here represents the frequency-shifted picture in Ref. 22, but the shifts (Eqs. (S7) and (S13)) are made slightly different from the original one in order to obtain the final expression in a symmetric form.

S3.2: A closed set of mean-field equations for the BEC–BCS-LASER crossover

By using the Hamiltonians presented above, within the HF approximation, the standard Green’s function and Heisenberg–Langevin approach yield the closed set of equations (1)-(7) in the main text. These equations are the same as obtained in Ref. 22 even though the expressions appear different^17^. In the derivation, it is assumed that a coherent photon field can be formed only in the ***q*** = 0 state

, (S15)

and that the system is in a steady state described by the polarization function *p****_k_***, the number of electrons *n*_e_,***_k_***, the number of holes *n*_h_,***_k_***, and the cavity photon field *a*_0_, respectively, which are defined as

, , , . (S16)

In Eqs. (1)–(7), the notation is transformed into the e–h picture

$\omega_{\text{e},\mathbf{k}}\equiv\omega_{\text{c},\mathbf{k}}$, , (S17)

with the Coulomb-renormalized dispersion relation

$\tilde{\omega}_{\alpha,\mathbf{k}}\equiv\omega_{\alpha,\mathbf{k}}+\Sigma_{\alpha,\mathbf{k}}^{\text{BGR}}$, , (S18)

where *α* ∈ {e, h}. Here, the unknown variables for the closed set of equations (1)–(7) are *a*_0_, *p****_k_***, *n*_e_,***_k_***, *n*_h_,***_k_***, and *μ*. Thus, we have shown a set of coupled equations. Eqs. (1)-(7), then, yields

, (S19)

. (S20)

when min[2*ħE****_k_***] ≳ *μ*_B_ – *μ* + 2*ħγ* + 2*k*_B_*T* by assuming *ω*_e_,***_k_*** = *ω*_h_,***_k_*** and *μ*_e_^B^ = *μ*_h_^B^ = *μ*_B_/2 for simplicity^22,16,17^. In this way, Eq. (S19) is reduced to the BCS gap equation for electrons and holes used in thermal equilibrium theories^20,21^. Therefore, in this situation, *μ* and min[2*ħE****_k_***] denote the chemical potential of the e–h–p system and the minimum energy required for breaking an e–h pair, respectively. Thus, the system can be well described by quasi-thermal equilibrium theories when the condition min[2*ħE****_k_***] ≳ *μ*_B_ – *μ* + 2*ħγ* + 2*k*_B_*T* is satisfied. This situation is already shown in Fig. S3A.

In contrast, non-equilibrium effects cannot be neglected for *μ*_B_ – *μ* ≳ min[2*ħE_k_*] – 2*ħγ* – 2*k*_B_*T*. Furthermore, Eqs. (1)–(7) result in

$0=-\text{i}\xi_{\text{ph,0}}a_{0}+\text{i}g\sum_{\mathbf{k}} p_{\mathbf{k}}-\kappa a_{0}$, (S21)

, (S22)

$0=-2\mathrm{Im}[\Delta_{\mathbf{k}}p_{\mathbf{k}}^{*}]-2\gamma[n_{\text{e}\left(\text{h}\right),\mathbf{k}}-f_{\text{e}\left(\text{h}\right),\mathbf{k}}]$, (S23)

when *μ*_B_ – *μ* ≳ 2*ħE****_k_*** + 2*ħγ* + 2*k*_B_*T* (which is ***k***-dependent) is satisfied. Again, for simplicity, it is assumed that *ω*_e_,***_k_*** = *ω*_h_,***_k_*** and *μ*_e_^B^ = *μ*_h_^B^. Here, *N****_k_*** ≡ *n*_e_,***_k_*** + *n*_h_,***_k_*** – 1 is the population inversion of the system and is the Fermi distribution function. Equations (S21)–(S23) are the very MSBE under the RTA used for steady-state semiconductor lasers^466^. Therefore, in this situation, *ħ*^–1^*μ* and Δ***_k_*** denote the laser oscillation frequency and the generalized Rabi frequency, respectively^26^. Thus, the ***k***-dependent condition *μ*_B_ – *μ* ≳ 2*ħE****_k_*** + 2*ħγ* + 2*k*_B_*T* determines the ***k***-region where use of the MSBE is justified. In other words, there are ***k***-regions described by the MSBE when *μ*_B_ – *μ* ≳ min[2*ħE****_k_***] + 2*ħγ* + 2*k*_B_*T*, and an example of this situation is shown in Fig. S3B. Here, we note that $\Delta_{\mathbf{k}}\cong g^{*}\sqrt{n}\cong2\Omega_{\text{R}}$ (*a*_0_ $\approx\sqrt{n}$) causes the exact Rabi splitting if there is no Coulomb term in the definition of , but Δ***_k_*** is also influenced by the BCS-like e–h Coulomb correlation term . In this sense, the energy gap (Fig. S2B and Fig. S3B) is formed not only by the standard Rabi splitting due to the strong field but also by the BCS-like e–h Coulomb correlations.

S3.3: Photoluminescence spectra

Based on standard quantum optics, the steady-state emission spectra observed outside the cavity can be described as

. (S24)

The emission spectra can then be divided into coherent and incoherent parts

$I_{\text{SS}}\left(\omega,\mathbf{q}\right)=I_{\text{SS}}^{\text{coh}}\left(\omega,\mathbf{q}\right)+I_{\text{SS}}^{\text{inc}}\left(\omega,\mathbf{q}\right)$, (S25)

with definitions of

$I_{\text{SS}}^{\text{coh}}\left(\omega,\mathbf{q}\right)\equiv2\kappa\left|a_{0}\right|^{2}\delta_{\mathbf{q},0}\delta\left(\omega\right)$, (S26)

, (S27)

where . Hence, the incoherent part of the emission spectra can be described by the photon Green’s functions in the frequency domain as

, (S28)

where *D*^R^, *D*^A^, and *D*^K^ are respectively the retarded, advanced, and Keldysh parts of the closed-time path of the (non-condensed) photon Green’s function defined as

, (S29)

where *T*_C_ is the time-ordering operator on the closed-time path^47,48^; *σ*_1_, *σ*_2_  ∈ {1, 2}; and the Heisenberg operators are

, . (S30)

In this context, the emission spectra can be discussed when *D*^R^, *D*^A^, and *D*^K^ in the frequency domain are obtained. Here, *D*^R^, *D*^A^, and *D*^K^ can be calculated if the self-energy Σ^ph^(*ω*; ***q***) for the photon Green’s function is determined because the Dyson equation for the photon Green’s function is described as

$D\left(\omega;\mathbf{q}\right)=[[D^{0}\left(\omega;\mathbf{q}\right)]^{-1}-\Sigma^{\text{ph}}\left(\omega;\mathbf{q}\right)]^{-1}$, (S31)

in the matrix form of

, (S32)

where *D*^0^, *D*, and Σ ^ph^ are described by *X* for notational simplicity. Therefore, in the following, we show the self-energy used for calculating *D*^R^, *D*^A^, *D*^K^ (Eq. (S31)), and $I_{\text{SS}}^{\text{inc}}\left(\omega,\mathbf{q}\right)$ (Eq. (S27)).

S3.4: Self-energies for the photon Green’s function

Photons in the cavity are basically influenced by the e–h system and by the free-space vacuum fields (photon bath), as shown in Fig. S5 and in the Hamiltonians in Subsection S3.1. As a result, the self-energy for the photon Green’s function in our study is written as

$\Sigma^{\text{ph}}\left(\omega;\mathbf{q}\right)=\Sigma_{\text{SR}}^{\text{ph}}\left(\omega;\mathbf{q}\right)+\Pi\left(\omega;\mathbf{q}\right)$, (S33)

$\Sigma_{\text{SR}}^{\text{ph}}\left(\omega;\mathbf{q}\right)$ and $\Pi\left(\omega;\mathbf{q}\right)$ are the self-energies due to the system–reservoir coupling (Eq. (S6)) and the light–matter coupling (Eq. (S12)), respectively. The latter describes the carrier-induced index change in conventional semiconductor laser theory^26^. By assuming that the photon-bath states are vacuum states, the self-energy $\Sigma_{\text{SR}}^{\text{ph}}$ can be written in the matrix form (Eq. (S32)) as

(S34)

within the approximation of Eq. (S8) (see also Fig. S8A). In contrast, the self-energy $\Pi$ can be written as

, (S35)

where $\tilde{K}_{0,\mathbf{q}}\left(\nu;\mathbf{k}_{1}\mathbf{k}_{2}\right)$ is a two-particle Green's function. In Eq. (S35), *g* is assumed to be real for simplicity, and $\tilde{K}_{0,\mathbf{q}}\left(\nu;\mathbf{k}_{1}\mathbf{k}_{2}\right)$ takes the same matrix form as Eq. (S32). Thus, it turns out that the two-particle Green’s function $\tilde{K}_{0,\mathbf{q}}\left(\nu;\mathbf{k}_{1}\mathbf{k}_{2}\right)$ is required to calculate the self-energy $\Pi\left(\omega;\mathbf{q}\right)$. However, in general, it is difficult to accurately obtain the two-particle Green’s function when the effects of Coulomb interactions are present. Therefore, in this study, the *T*-matrix approximation is applied, as shown in Fig. S8,

$\tilde{K}_{0,\mathbf{q}}\left(\omega;\mathbf{k}_{1}\mathbf{k}_{2}\right)=-K_{0,\mathbf{q}}\left(\omega;\mathbf{k}_{1}\mathbf{k}_{2}\right)-K_{0,\mathbf{q}}\left(\omega;\mathbf{k}_{1}\mathbf{k}_{1}\right)T\left(\omega;\mathbf{k}_{1}\mathbf{k}_{2}\right)K_{0,\mathbf{q}}\left(\omega;\mathbf{k}_{4}\mathbf{k}_{2}\right)$, (S36)

, (S37)

where ***I***_4×4_ is the unit 4×4 matrix and *K*_0_***_,q_***(*ω*;***k***_1_***k***_2_) is the two-particle Green’s function without any interactions. Therefore, in Eqs. (S36) and (S37), the matrix elements of *K*_0_***_,q_***(*ω*;***k***_1_***k***_2_) can be described by using the one-particle Green’s functions

,

(S38)

,

(S39)

where *α*_1_, *α*_2_ ∈ {1, 2}, $\bar{\alpha}_{i}=2, 1$ for $\alpha_{i}=1, 2$, and the one-particle Green’s functions can be written as

, (S40)

, (S41)

in the matrix form of

$G^{\text{R/A/K}}\left(\nu;\mathbf{k}\right)\equiv\left(\begin{matrix} G^{\text{R/A/K}}\left(\nu;11;\mathbf{k}\right) & G^{\text{R/A/K}}\left(\nu;12;\mathbf{k}\right) \\ G^{\text{R/A/K}}\left(\nu;21;\mathbf{k}\right) & G^{\text{R/A/K}}\left(\nu;22;\mathbf{k}\right) \end{matrix}\right)$. (S42)

These Green’s functions (Eqs. (S40) and (S41)) were already obtained from the solutions of Eqs. (1)–(7). As a result, the *T* matrix and the two-particle Green’s function can be calculated with Eqs. (S36) and (S37). Hence, the self-energy $\Pi\left(\omega;\mathbf{q}\right)$ can be calculated. Thus, at this stage, the formulations for the emission spectra are complete. For readers unfamiliar with Green’s functions, the procedures for calculating the emission spectra are given in Subsection S3.5, where the parameters used in our study are also described.

Section S3.5: Procedures and parameters for calculating emission spectra

The formalism for calculating the emission spectra has been presented in Subsections S3.3 and S3.4. The procedures for calculating the emission spectra are now summarized as follows:

**Step 1**: Solve the simultaneous system of equations consisting of Eqs. (1)–(7) for the unknown variables *a*_0_, *p****_k_***, *n*_e_,***_k_***, *n*_h_,***_k_***, and *μ*.

**Step 2**: Evaluate the one-particle Green’s functions (Eqs. (S40) and (S41)) by using the values of *a*_0_, *p****_k_***, *n*_e_,***_k_***, *n*_h_,***_k_***, and *μ* obtained in Step 1.

**Step 3**: Evaluate the two-particle Green’s function *K*_0_,***_q_***(*ω*;***k***_1_***k***_2_) (Eqs. (S38) and (S39)) by using the one-particle Green’s functions obtained in Step 2.

**Step 4**: Find the *T* matrix satisfying Eq. (S37) for *K*_0_,***_q_***(*ω*;***k***_1_***k***_2_) obtained in Step 3.

**Step 5**: Calculate the two-particle Green’s function $\tilde{K}_{0,\mathbf{q}}\left(\omega;\mathbf{k}_{1}\mathbf{k}_{2}\right)$ in Eq. (S36) by using the *T* matrix obtained in Step 4.

**Step 6**: Calculate the self-energy Σ^ph^(*ω*; ***q***) for the photon Green’s function (Eq. (S33) with Eqs. (S34) and (S35)) by using $\tilde{K}_{0,\mathbf{q}}\left(\omega;\mathbf{k}_{1}\mathbf{k}_{2}\right)$ obtained in Step 5.

**Step 7**: Calculate the photon Green’s function *D*(*ω*; ***q***) by using Eq. (S31) and the value of Σ^ph^(*ω*; ***q***) obtained in Step 6.

**Step 8**: Calculate the emission spectra $I_{\text{SS}}^{\text{inc}}\left(\omega,\mathbf{q}\right)$ by using the photon Green’s function obtained in Step 7.

By following Steps 1−8, the spectra $I_{\text{SS}}^{\text{inc}}\left(\omega,\mathbf{q}=0\right)$ in Fig. 5 in the main text were obtained. In our calculations, the contact potential model *U****_q_*** = *U* is used for simplicity, and therefore, the *k* dependence of Δ*_k_* is eliminated as Δ*_k_* = Δ. The value of *U* = 2.66 × 10^-10^ eV is determined for the (1S) exciton level to be located at 10 meV less than the band-gap energy *E*_g_ = 1.5 eV with a cut-off wavenumber *k*_c_ = 1.36 × 10^9^ m^-1^. The cavity mode for ***q*** = 0 is on resonance with the (1S) exciton level (*E*_cav_ = *E*_g_ – 10 meV), and the lower polariton level is formed at an energy of 10 meV below the cavity energy when using *ħg* = 6.30 × 10^-7^ eV. In addition, *ħω*_e_,***_k_*** = *ħω*_h_,***_k_*** = *ħ*^2^*k*^2^/2*m* + *E*_g_/2 (*m* = 0.068 *m*_0_, where *m*_0_ is the free-electron mass) and *T* = 10 K are assumed with charge neutrality *μ*_e_^B^ = *μ*_h_^B^. In this context, we note that the calculations are qualitative even though the parameters are determined as realistically as possible. For the other parameters, *ħκ* = 100 μeV and *ħγ* = 4 meV are used.

**Fig. S1.**

Dressed states and their spectra (**A**) for an atom with a strong field and (**B**) for an atom strongly coupled with a cavity.

**Fig. S2.**

Energy diagram in a high-density regime. (**A**) The effects of the electron–electron and hole–hole Coulomb interactions are considered, but the dipole coupling to the cavity photons and the e–h attractive Coulomb interactions are neglected. (**B**) The effects of the dipole coupling and the e–h Coulomb interaction are also considered. In this case, the electron band (the solid blue curve) is mixed with the +*ħω*_0_-shifted hole band (the dashed red curve). In the same manner, the hole band (the solid red curve) is mixed with the –*ħω*_0_-shifted electron band (the dashed blue curve). Here, the contact potential *U'****_q_***_−_***_k_*** = *U* is assumed, and Δ***_k_*** = Δ.

**Fig. S3.**

Energy diagram under thermal equilibrium and non-equilibrium conditions. These figures are the same as Fig. 3A and 3B in the main text.


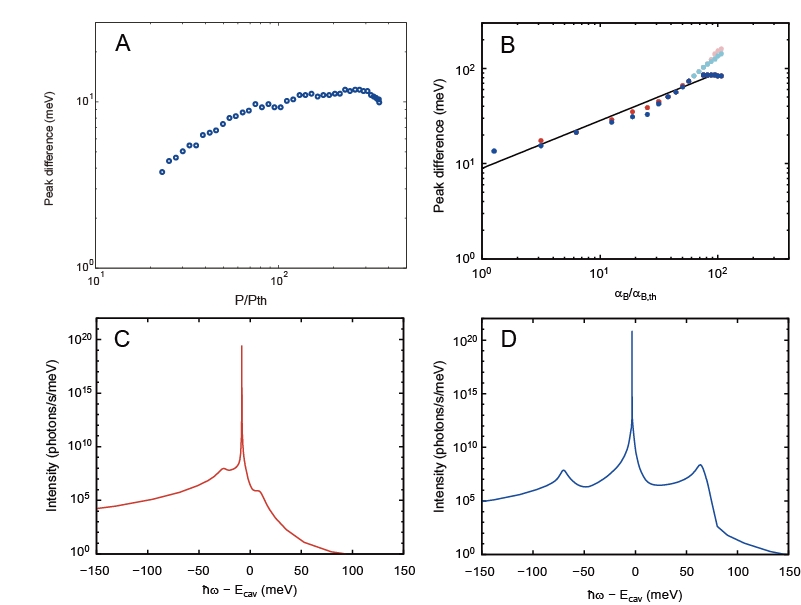


**Fig. S4.**

Experimental (panel (**A**)) and theoretical (panel (**B**)) results of the peak differences as a function of the pumping. Note that panel (A) is reprinted as Fig. 2 (D) while panel (B)-(D) are reprinted as Fig. 5. In panel (B), the blue and red data points are the peak energies for the high- and low-energy peaks, respectively, which correspond to the transitions represented by the blue and red arrows in Fig. S3. In the lasing condition, the additional data points in aqua and pink correspond to the aqua and pink transitions in Fig. S3, respectively. The black lines of panels (A) and (B) are proportional to the square root of the pump power *P* normalized to *P*_th_ and *α*_B_ ≡ *μ*_B_ – *E*_LP_ normalized to *α*_B,th_ ≡ *μ*_B,th_ – *E*_LP_ = 1.64 meV with the lower polariton energy *E*_LP_ with zero detuning, respectively. The corresponding theoretical treatment is in Section S3. (**C** and **D**) Calculated photoluminescence spectrum $I_{\text{SS}}^{\text{inc}}\left(\omega,\mathbf{q}=0\right)$ at *μ*_B_ – *E*_LP_ = 5 meV ((C), low excitation density) and 80 meV ((D), high excitation density) indicated by arrows in Fig. 4B in the main text. The experimental peak differences are an order of magnitude smaller than the calculated values. This discrepancy may be caused by dephasing and polariton–polariton scattering processes not taken into account in our theory.

**Fig. S5.**

Schematic diagram of the system studied in this section (Section S3), showing *n* equivalent two-level systems coupled with the cavity.

**Fig. S6.**

Results calculated with the TLS model. (**A**) The number of photons inside the cavity as a function of the pumping parameter *α*_B_ ≡ *μ*_B_ – *E*_LP_ normalized to *α*_B,th_ ≡ *μ*_B,th_ – *E*_LP_ = 3.88 meV. Here, red data points and curves are used to indicate when the system can still be regarded as in a quasi-equilibrium state, and otherwise blue plot elements are used. For comparison, a result for the thermal equilibrium limit is also displayed by the solid black line (*κ* = 0 and *γ* = 0^+^). The gray dotted line represents the bare cavity energy *E*_cav_ (= *ħω*_x_). (**B** and **C**): Calculated photoluminescence spectra $I_{\text{SS}}^{\text{inc}}\left(\omega,\mathbf{q}=0\right)$ at *α*_B_ = 20 meV (B) and 120 meV (C), which correspond to the data points indicated by the arrows in panel (A). The gray dashed line in panel (C) is a guide for the eyes to help compare the sideband peak intensities.

**Fig. S7.**

The model of the electron–hole–photon system.

**Fig. S8.**

Self-energy diagrams with the *T*-matrix approximation. (**A**) $\Sigma_{\text{SR}}^{\text{ph}}$ and (**B**) Π are the self-energies due to the system–reservoir coupling and the light–matter coupling, respectively. (**C**) $\tilde{K}_{0,\mathbf{q}}$ and (**D**) *T* are the two-particle Green’s function and *T* matrix, respectively. The double and wavy-dashed lines represent the electronic Green’s function and photon-bath Green’s functions, respectively. The gray wavy line represents the photon Green’s function that will be connected to the self-energy diagrams. The dashed line corresponds to the Coulomb interaction.


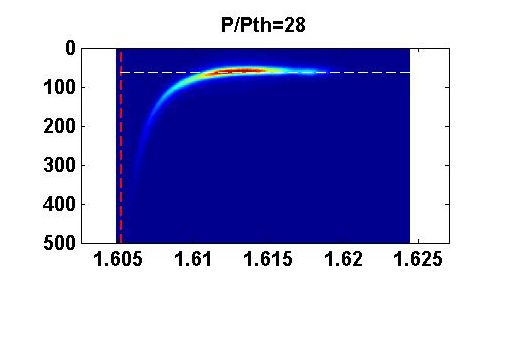


**Fig. S9.**

Energy relaxation in a high pump power regime (28*P_th_*). Though the pump laser power is lower than that of Fig. 2A (75*P_th_*), the behaviour of the relaxation into lower polariton energy far below *P_th_* shown by red dotted line is the same. At this pump power, the high-energy peak is still close to and not completely separated from the main peak as also shown in Fig. 2C.

**References**

1. Szymanska, M. H. Keeling, K. and Littlewood, P. B., Mean-field theory and fluctuation spectrum of a pumped decaying Bose-Fermi system across the quantum condensation transition. *Phys*. *Rev*. *B* **75**, 195331 (2007).
2. Keeling, J. Szymanska, M. H. and Littlewood, P. B., *Optical Generation and Control of Quantum Coherence in Semiconductor Nanostructures*, G. Slavcheva, P. Roussignol, Eds. (Springer, Berlin, 2010), *chap. 12*.
3. Wouters, M. and Carusotto, I. Excitations in a Nonequilibrium Bose-Einstein Condensate of Exciton Polaritons. *Phys*. *Rev*. *Lett*. **99**, 140402 (2007).
4. Chow, W. W. *et* *al*., Nonequilibrium Model for Semiconductor Laser Modulation Response. IEEE *J*. *Quantum Electron*. **38**, 402 (2002).
5. Rammer, J. *Quantum Field Theory of Non-equilibrium States* (Cambridge University Press, New York, 2007).
6. Kamenev, A. *Field Theory of Non-Equilibrium Systems* (Cambridge University Press, New York, 2011).
